# An R package implementation of multifactor dimensionality reduction

**DOI:** 10.1186/1756-0381-4-24

**Published:** 2011-08-16

**Authors:** Stacey J Winham, Alison A Motsinger-Reif

**Affiliations:** 1 Department of Statistics, North Carolina State University, Raleigh NC 27695, USA; 2 Department of Health Sciences Research, Mayo Clinic, Rochester MN 55905, USA; 3 Bioinformatics Research Center, North Carolina State University, Raleigh NC 27695, USA

## Abstract

**Background:**

A breadth of high-dimensional data is now available with unprecedented numbers of genetic markers and data-mining approaches to variable selection are increasingly being utilized to uncover associations, including potential gene-gene and gene-environment interactions. One of the most commonly used data-mining methods for case-control data is Multifactor Dimensionality Reduction (MDR), which has displayed success in both simulations and real data applications. Additional software applications in alternative programming languages can improve the availability and usefulness of the method for a broader range of users.

**Results:**

We introduce a package for the R statistical language to implement the Multifactor Dimensionality Reduction (MDR) method for nonparametric variable selection of interactions. This package is designed to provide an alternative implementation for R users, with great flexibility and utility for both data analysis and research. The 'MDR' package is freely available online at http://www.r-project.org/. We also provide data examples to illustrate the use and functionality of the package.

**Conclusions:**

MDR is a frequently-used data-mining method to identify potential gene-gene interactions, and alternative implementations will further increase this usage. We introduce a flexible software package for R users.

## Background

With advances in genotyping technologies, a breadth of high-dimensional data is now available with unprecedented numbers of genetic markers to perform association mapping in human genetics. Identifying variants associated with complex human traits is a common problem and data-mining approaches to variable selection are frequent methods of analysis. There is growing evidence that epistasis may play a role in disease risk, and many variable selection approaches have been developed to consider potential gene-gene and gene-environment interactions. One of the most commonly used techniques for case-control data is Multifactor Dimensionality Reduction (MDR), a nonparametric exhaustive search method that considers all combinations of potentially interacting loci and classifies individuals to disease status based on their genetic information [1]. MDR has been highly successful in human genetics, with a large number of associations identified in real data applications; additionally, the performance of the method has been extensively studied in a range of simulation experiments and has undergone numerous developments and extensions to improve performance [2,3].

Currently software is available which implements the MDR method, including a GUI implementation available at http://www.epistasis.org[4]; however, additional implementations in alternative programming languages are welcome in order to improve the widespread usability of the method for a broader range of users. The free and open-source R statistical software is one of the most widely-used statistical software environments. We introduce a new package for the R statistical language, 'MDR'. The package is designed to provide an alternative implementation for R users, and has great flexibility and utility for both data analysis and research. Currently, an R package exists to implement a parametric extension, model-based MDR ('mbmdr') [5], however, not in the original nonparametric form that is most commonly used and without extensive flexibility and analysis options. The package 'MDR' implements MDR for variable selection of interactions as first outlined in [1] and described in more detail in [4], providing options for internal validation and functions to summarize the fit and perform post-hoc inference, and is available at http://www.r-project.org/.

In its traditional implementation, MDR is considered both statistically and genetically non-parametric because it does not estimate any statistical model parameters or assume a particular genetic inheritance mode [1]. MDR reduces the dimensionality of the data by viewing combinations of loci (that may interact) as a series of multi-factorial genotypes, rather than as separate variables. MDR creates a classification rule based on these combinations using a Naïve Bayes classifier, assigning genotype combinations with a large ratio of cases to controls as high-risk and low-risk otherwise [6]. Using this high-risk/low-risk parameterization, a measure of the accuracy of the classification rule is evaluated, which is typically some measure of classification accuracy, the proportion of correctly classified individuals. A final model is chosen to maximize this accuracy, or to misclassify the fewest number of individuals. The final model will also perform well in terms of prediction, and internal validation measures such as cross-validation measure prediction accuracy [7]. It is this traditional implementation that we employ in the R package 'MDR'.

## Implementation

This package utilizes balanced accuracy (BA) as the evaluation measure for comparing different combinations of variables, defined as

$$BA=\frac{1}{2}\left(\frac{TP}{TP+FN}+\frac{TN}{TN+FP}\right)$$

where (*TP, TN, FP, FN*) represent the number of true positives, true negatives, false positives, and false negatives classified by a particular combination of loci, respectively. Balanced accuracy, the arithmetic mean of sensitivity and specificity, has been shown to outperform the traditional measure of classification accuracy when datasets are unbalanced [8]. Other evaluation measures are possible, including additional contingency table measures [9], but are not currently included in this package.

This package assumes binary case-control data with categorical predictor variables. The binary response variable is coded as 0 or 1, and the categorical predictors (typically SNP genotypes) are coded numerically (0, 1, 2, etc.). The user can specify the particular genotype encoding. Additionally, the threshold for assigning high-risk/low-risk status to variable combinations can also be controlled by the user.

### Internal Validation

This package provides a base function 'mdr' to fit a list of MDR models, ranked with balanced accuracy. However, in all data-mining methods, over-fitting a model to a particular data set is a concern and it is suggested that MDR be implemented in conjunction with an internal validation technique. This package provides two such procedures: *k*-fold cross-validation and three-way split internal validation.

In *k*-fold cross-validation, the data are randomly split into *k *equal intervals, where *k*-1 intervals are used for training and one interval is used for testing [7]. The best MDR model is determined from the training set for each size of interaction and an estimate of the model's prediction accuracy is calculated from the testing set. This procedure is repeated for all *k *possible splits of the data and a final model is chosen to maximize both prediction accuracy and cross-validation consistency across each split. The function 'mdr.cv' implements cross-validation and allows the user to specify the highest level of interaction to consider, as well as the number of intervals *k*; typically a value of *k *= 5 or 10 yields high performance [7].

In three-way split internal validation, the data are randomly split into three sets for training, testing, and validation [10]. MDR is first implemented in the training set for all possible combinations of loci and the *x *models with the highest balanced accuracy are retained for evaluation in the testing set. MDR is next performed on all *x *models in the testing set and the best model for each level of interaction is preserved for evaluation of predictive ability in the validation set. A final model is chosen to maximize balanced accuracy in the validation set. The function 'mdr.3WS' implements three-way split internal validation and allows the user to specify the ratio of the three data splits (training:testing:validation), and also the number of potential models *x *from the training set to be evaluated in the testing set.

Both internal validation methods create objects of class 'mdr', a list of the final selected model loci and its prediction accuracy, the top models and their prediction accuracies, and the high-risk/low-risk characterization of the final model.

### Methods

Three methods exist for objects of class 'mdr': 'summary', 'plot', and 'predict'. The 'summary' method provides a table summarizing the model fit at each stage of interaction. The 'plot' method provides a contingency table of bar graphs for the final model, portraying the numbers of cases and controls in each genotype combination, similar to the GUI implementation at http://www.epistasis.org. The 'predict' method allows the user to predict case-control status on a new, independent set of data with a model obtained from a previously fit 'mdr' object.

### Post-hoc Functions for Inference

After an MDR model has been fit, a number of functions exist for inference on that fit. Permutation testing is available to test the significance of the reported measure of prediction accuracy; case-control status is randomly permuted a number of times (specified by the user), and the resulting prediction accuracies from each MDR fit of the permuted data sets are compared to a specified accuracy [11]. In addition to the traditional permutation test of the full MDR model, we also incorporate a permutation test of interaction based on the likelihood ratio test, as described in Edwards et al [12]. Additionally, estimates of prediction accuracy are obtained from retrospective case-control data, and therefore may not reflect the true accuracy of prospective predictions. Using a previously estimated population prevalence rate provided by the user, these prediction accuracy estimates can be adjusted using one of two available post-hoc procedures implemented in 'boot.error' and 'mdr.ca.adj' [13].

## Results and Discussion

To illustrate the usage of the package, we provide a computational example using a simulated dataset of 250 individuals who were genotyped at 25 SNPs. We first fit an MDR model using cross-validation with cv = 5 cross-validation intervals. We consider all combinations of SNPs up to size K = 3 and the default settings for the other options and then summarize the fit:

> library(MDR)

> data(mdr1)

> fit.cv<-mdr.cv(data = mdr1, K = 3, cv = 5, ratio = NULL,

equal = "HR", genotype = c(0, 1, 2))

> summary(fit.cv)

From Table 1 below we see that the MDR fit identified the two-way model of SNPs 4 and 9 as the best model predictive of disease status. This model minimized balanced accuracy in 5 out of 5 cross-validation intervals and estimates a prediction accuracy of 64.12%.

**Table 1:** Summary table for MDR fit with 5-fold cross-validation

**Level**		**Best** **Models**	**Classification Accuracy**	**Prediction Accuracy**	**Cross-Validation Consistency**
	1	9	61.77	60.95	4
*	2	4 9	67.24	64.12	5
	3	4 6 9	72.89	61.80	2

'*' indicates overall best model

We can also fit an MDR model using three-way split internal validation, also allowing for combinations of SNPs up to size K = 3 and the default settings for the other options, and then summarize the fit:

> fit.3WS<-mdr.3WS(data = mdr1, K = 3, × = NULL, proportion = NULL, ratio = NULL, equal = "HR", genotype = c(0, 1, 2))

> summary(fit.3WS)

Unlike the cross-validation fit, the MDR fit using three-way split validation identified a larger three-way model of SNPs 4, 9, and 24 as the best model predictive of disease status, which maximizes balanced accuracy in the validation set (Table 2). This model estimates predictive ability with a validation accuracy of 73.03%. Notice, however, that the results for the best two-way model, SNPs 4 and 9, are similar to the results of the cross-validation fit.

**Table 2:** Summary table for MDR fit with three-way split validation

**Level**		**Best** **Models**	**Training Accuracy**	**Testing Accuracy**	**Validation Accuracy**
	1	4	56	67.95	53.78
	2	4 9	67	71.79	65.86
*	3	4 9 24	74	78.61	73.03

'*' indicates overall best model

**Figure 1:**
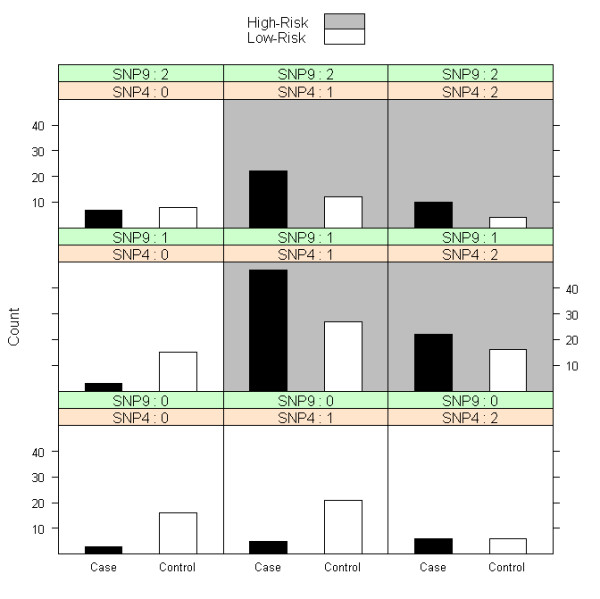
**The result of a sample call to 'plot' after an MDR fit with 5-fold cross-validation on a simulated dataset with 250 individuals genotyped at 25 SNPs**.

**Figure 2:**
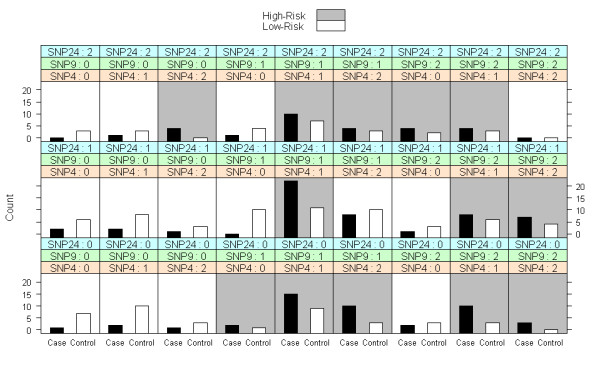
**The result of a sample call to 'plot' after an MDR fit with three-way split on a simulated dataset with 250 individuals genotyped at 25 SNPs**.

After each MDR fit, we can visually display the results for the MDR final model, including the particular pattern of interaction, using the method 'plot' (Figures 1 and 2). We can see which genotype combinations are high-risk for the cross-validation (Figure 1) and three-way split (Figure 2) examples discussed here. Additionally, we can perform other measures of post-hoc inference. For instance, suppose we are interested in making disease predictions from the model fit with cross-validation. We can predict disease status on a new, independent set of genotype data using the method 'predict', and evaluate the quality of these predictions with our prediction error/accuracy estimate. However, the dataset 'mdr1' was balanced with 125 cases and 125 controls, reflecting a retrospective association study. Likely, disease prevalence is much less than 0.50 and we can use this knowledge to adjust our estimates of prediction error. Suppose the simulated dataset is from a common, complex disease with a prevalence rate of 0.10. We can update our prediction error estimate from the cross-validation fit using bootstrap resampling (with b = 100 samples) or an algebraic adjustment as follows:

> boot.error(mdr1,prev = 0.10, model = fit.cv$'final model', hr = fit.cv$'high-risk/low-risk', b = 100)

$'classification error estimate'

[1] 47.792

$'classification accuracy estimate'

[1] 52.208

> mdr.ca.adj(mdr1, model = fit.cv$'final model', hr = fit.cv$'high-risk/low-risk', prev = 0.10)

$'adjusted classification accuracy'

[1] 51.76

$'adjusted classification error'

[1] 48.24

After the prospective adjustment, we now estimate a prediction accuracy of around 52%, a reduction from the original retrospective estimate of 64.12%.

### Computation Time

An important aspect of any new software package is computation time. We evaluate the run time of our MDR software on datasets of three different sizes to provide the user with benchmarks in terms of computation time. Dataset MDR1 contains a sample of size *n *= 250 with *p *= 25 loci, dataset MDR2 contains a sample of size *n *= 250 with *p *= 50 loci, and dataset MDR3 contains a sample of size *n *= 500 with *p *= 50 loci. MDR with both 5-fold cross-validation and 3WS internal validation were performed using the described R package. Default settings were used for all parameters and combinations of loci up to *K *= 3 were considered. Results are also compared with the Java GUI version in Table 3. Results were executed on a PC with a 2.8 GHz dual-core processor. Run time increases moderately with sample size and significantly with the number of loci, and 3WS internal validation is considerably faster than CV. Additionally, the functions of 'MDR' are much slower than the Java GUI implementation.

**Table 3:** Sample run time in seconds for the package 'MDR' and for the GUI version

Time (seconds)	'MDR' 5-fold CV	'MDR' 3WS	GUI
**MDR1 (*n *= 250, p = 25)**	221.85	41.87	1.253
**MDR2 (*n *= 250, *p *= 50)**	1951.05	345.67	3.902
**MDR3 (*n *= 500, *p *= 50)**	2138.25	375.42	6.329

The R computing environment is known to be much slower than competing languages such as C++ and Java, so the increased run-time as compared to the Java GUI implementation is not surprising or unreasonable (see http://dan.corlan.net/bench.html). Increased computation time, particularly for high-dimensional data is a limitation of R as compared to other programming languages. While a traditional R package cannot compete with Java or C++ in terms of computation time, reducing computation time is possible. For instance, parts of the R package source code could be written in C. Furthermore, because many of the calculations of MDR are independent, many of the looping constructs could be executed in parallel. Great strides have recently been made in the areas of parallel computing in R, and this package could be extended to include parallelization using a number of recently developed packages such as 'foreach', 'doMC', and 'doSNOW' (see http://cran.r-project.org/web/views/HighPerformanceComputing.html). The use of parallel computing could drastically reduce computation time for MDR, particularly on a cluster machine. Because of the variation in R usage on single workstations, multiple workstations, and multi-node clusters, parallelization is not currently implemented in this package. Additionally, there are memory limitations to R in terms of high-dimensional datasets, which are typically experienced with genetic data. Advances have been made in terms of increased memory, and the 'bigmemory' package allows the user to store and analyze large datasets. The open source nature of the R environment and this package allow this flexibility for these types of extensions.

Due to these limitations in the current implementation, without the aforementioned extensions, the usefulness of this package is primarily reserved for smaller candidate gene analysis and/or searches for low order models in larger scale candidate gene searches in real data as well as methodological research. In real data analysis, the package is most suitable for a moderate number of loci to evaluate candidate interactions rather than a genome-wide variable selection. Moreover, the R implementation allows the user to integrate this data-mining analysis into more traditional statistical analyses. In addition, because it's written in such a flexible environment, the package allows for easy extension of the MDR methodology for further research.

## Conclusions

We introduce new software to implement the MDR method for variable selection of epistatic interactions using the R statistical language. The package 'MDR' is designed to provide an alternative implementation for R users, with great flexibility and utility for both data analysis and research.

## Availability and Requirements

**Project name: **R package, MDR

**Project home page**: http://cran.r-project.org/web/packages/MDR/index.html

**Operating systems: **Linux, Mac OS, Windows

**Programming language: **R

**Other requirements: **R package, lattice

**License: **GNU GPL-2

Any restrictions to use by non-academics:

## Competing interests

The authors declare that they have no competing interests.

## Authors' contributions

SJW programmed and designed the R package and contributed to writing the manuscript. AMR facilitated with the design of the R package and contributed to writing the manuscript. All authors read and approved the final manuscript.
